# New insights in acetaminophen toxicity: HMGB1 contributes by itself to amplify hepatocyte necrosis in vitro through the TLR4-TRIF-RIPK3 axis

**DOI:** 10.1038/s41598-020-61270-1

**Published:** 2020-03-27

**Authors:** Charlotte Minsart, Claire Liefferinckx, Arnaud Lemmers, Cindy Dressen, Eric Quertinmont, Isabelle Leclercq, Jacques Devière, Richard Moreau, Thierry Gustot

**Affiliations:** 1 Laboratory of Experimental Gastroenterology, Université Libre de Bruxelles, Brussels, Belgium; 2 Department of Gastroenterology, HepatoPancreatology and Digestive Oncology, C.U.B. Erasme Hospital, Université Libre de Bruxelles, Brussels, Belgium; 3 Laboratory of Physiology and Pharmacology, Université Libre de Bruxelles, Brussels, Belgium; 4 Laboratory of Hepato-Gastroenterology, Institut de Recherche Expérimentale et Clinique, Université Catholique de Louvain, Brussels, Belgium; 5 Inserm Unité 1149, Centre de Recherche sur l’inflammation [CRI], Paris, France; 6 UMR S_1149, Université Paris Diderot, Paris, France; 7 DHU UNITY, Service d’Hépatologie, Hôpital Beaujon, APHP, Clichy, France

**Keywords:** Cell signalling, Mechanisms of disease

## Abstract

Extracellular release of HMGB1 contributes to acetaminophen-induced liver injury. HMGB1 acts as a danger-associated molecular patterns during this toxic process but the mechanisms of action and targeted cells are incompletely defined. Here we studied, *in vitro*, the role of HMGB1 in amplifying the acetaminophen-induced hepatocyte necrosis process. Using cultured HepaRG cells, primary human hepatocytes and selective chemical inhibitors we evaluated acetaminophen-induced toxicity. We confirmed that addition of acetaminophen induced HepaRG cell death and HMGB1 release. We showed that inhibition of HMGB1 decreased acetaminophen-induced HepaRG cell death, suggesting a feedforward effect. We provide the first evidence that exposure of HepaRG cells to recombinant human HMGB1 (rhHMGB1) also resulted in cell death. Moreover, we found that both acetaminophen and rhHMGB1 induced programmed HepaRG cell necrosis through a RIPK3-dependent mechanism. By using TLR4 blocking antibody, we demonstrated the reduction of the HepaRG cell death induced by acetaminophen and rhHMGB1. Furthermore, inhibition of TRIF, known to induce a RIPK3-dependent cell death, reduced rhHMGB1-induced HepaRG cell death. Our data support that released HMGB1 from acetaminophen-stressed hepatocytes induced necrosis of neighboring hepatocytes by TLR4-TRIF-RIPK3- pathway. This *in vitro* study gives new insights in the role of HMGB1 in the amplification of acetaminophen-induced toxicity.

## Introduction

Voluntary or accidental acetaminophen (N-acetyl-p-aminophenol, APAP) overdose remains the first cause of acute liver failure in western countries, accounting for up to 60–70% of patients in some observations^1,2^. Mechanisms of hepatotoxicity are incompletely understood. The mechanism of acetaminophen toxicity has been well studied. Following ingestion, a majority (>90%) of acetaminophen is metabolized by glucuronidation and sulfation reactions to produce non-toxic metabolites. A small fraction (<10%), undergoing oxidation and acetaminophen is metabolized by CYP450 isoforms, mainly CYP2E1, to N-acetyl-p-benzoquinone imine (NAPQI), a toxic metabolite. Under normal conditions NAPQI, binding covalently to cysteine groups on proteins (APAP adducts), is rapidly detoxified by glutathione. With acetaminophen toxicity, cellular glutathione is depleted resulting of the accumulation of APAP adducts, mainly with mitochondrial protein, inducing oxidative stress and mitochondrial injuries that lead to centrilobular hepatocyte necrosis and liver failure^3–6^.

A release of danger-associated molecular patterns (DAMPs) by dying hepatocytes during APAP-induced liver injury was demonstrated in both murine models and patients. The current paradigm suggests that cell death leads to the release of DAMPs alerting immune cells and triggering a self-amplifying circuit resulting to tissue damage and finally organ failure^7^. However, the current knowledge on those DAMPs that activate hepatic inflammatory cells is controversial.

High-mobility Group Box 1 (HMGB1) is a DAMP originating from the nucleus and released during the necrotic process^8^. HMGB1 protein is a small ubiquitous non-histone nuclear protein that acts as an architectural chromatin-binding factor involved in the maintenance of nucleosome structure and regulation of gene transcription by binding the minor groove of DNA^9,10^.

Two types of cells that can release HMGB1 have been reported. Firstly, immune cells (such as macrophages, mature dendritic cells, and natural killer cells) can actively secrete HMGB1 as part of the inflammatory response^11,12^. Secondly, necrotic cells can also release HMGB1. Although HMGB1 is a nuclear protein, cytoplasmic HMGB1 can be detected in necrotic cells and it can be released into the extracellular space by cellular damage^13^. Serum HMGB1 levels are increased in humans APAP hepatotoxicity and correlated with the severity of liver failure^14,15^. In mice, HMGB1 blockade significantly reduces the severity of APAP-induced hepatotoxicity^16,17^.

The mechanism of action of HMGB1 in APAP-induced liver injury remains unclear. In this study, we hypothesized that HMGB1, released during the hepatotoxic process induced by APAP, could act directly on hepatocytes independently of any intervention by inflammatory cells.

To investigate this hypothesis, *in vitro* models are indispensable tools. In this study, HepaRG cells were used. HepaRG cell line is an immortalized hepatic cell line who exhibit many characteristics of primary human hepatocytes including morphology, expression of key metabolic enzymes, expression of nuclear receptors, and drug transporters. Thanks to all these features, HepaRG cells are a recognized tool to investigate APAP hepatotoxicity^18–20^.

## Results

### HMGB1 is released by APAP-damaged hepatocytes

**Figure 1:**
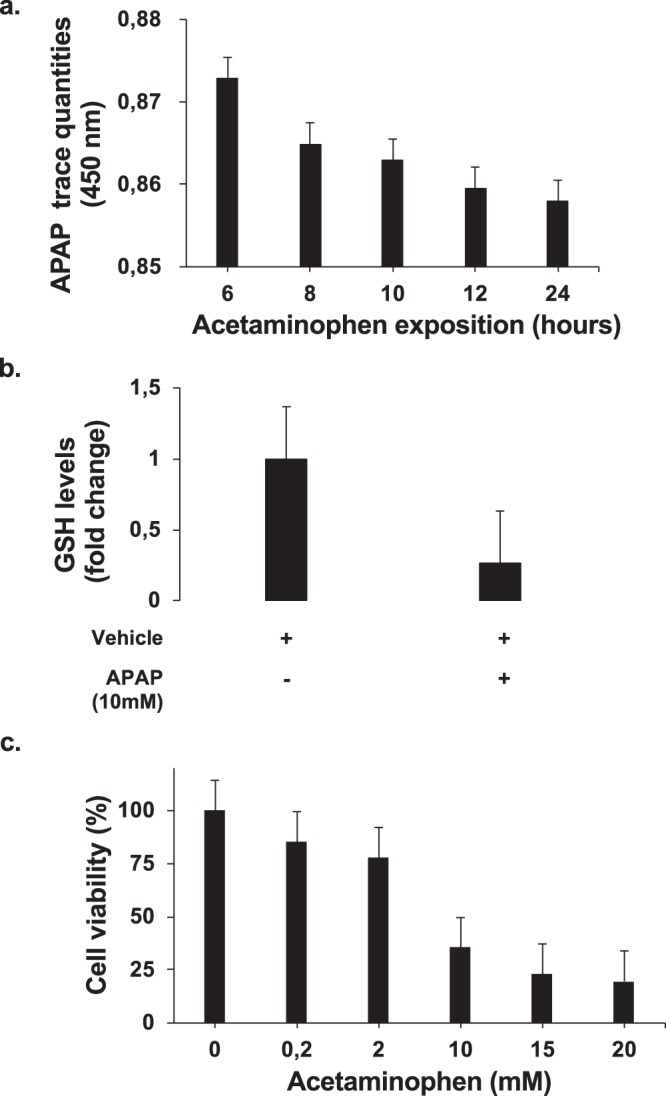
APAP metabolism and hepatotoxicity. (**a**) Measurement of acetaminophen in the supernatant of HepaRG cells treated with 10 mM APAP at increasing time points (0–24 hours). * p < 0.05 vs 6 h time point. (**b**) HepaRG cells were treated with APAP (10 mM) for 24 hours and GSH levels were measured. The enzyme concentration obtained is expressed as nanomoles of enzyme per milligram of protein using bovine serum as a standard. ** p < 0.01 vs vehicle. (**c**) HepaRG cells were exposed to increasing concentrations of APAP (0–20 mM) and cell viability was assessed by MTT (expressed as a percentage of unexposed cells (0)) at 24 hours. Results are expressed as mean ± SEM. ** vs. 0, p < 0.01. Experiments were reproduced three times.

As previously shown, HepaRG cells were able to metabolize APAP. Indeed, a time-dependent decrease of APAP trace quantities in the supernatant (reflected by absorbance decrease) and reduced glutathione (GSH) levels in HepaRG cells at 24 hours of APAP exposure confirmed it (Fig. 1a,b). Moreover, a significant dose-dependent HepaRG death was observed from 10 mM of APAP with a cell viability percentage of 35.4 ± 3.34% (p < 0.001) (Fig. 1c).

**Figure 2:**
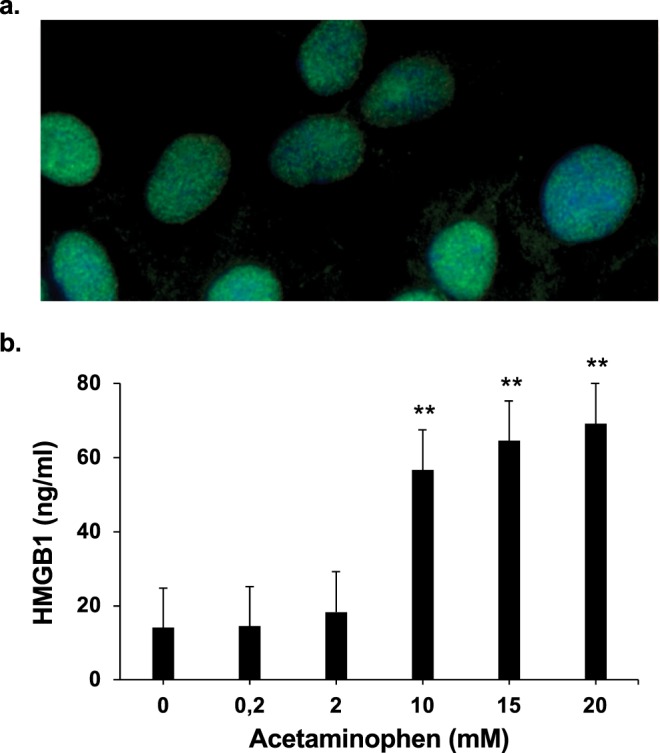
Cellular location of HMBG1 and APAP-induced HMBG1 release. (**a**) Immunofluorescence of HMGB1 was performed on HepaRG cells untreated (x600 magnification). Green, HMGB1; Blue, DAPI. (**b**) HMGB1 levels were measured by ELISA assay in the supernatant of HepaRG cells exposed to increasing concentrations of APAP (0–20 mM) for 24 hours. Results are expressed as mean ± SEM. ** vs. 0, p < 0.01. Experiments were reproduced three times.

HMGB1 is expressed in the nucleus of HepaRG cells (Fig. 2a) and its release in the supernatant was observed when the cells were exposed to APAP for 24 hours in a dose-dependent manner as shown in Fig. 2b (significantly (p = 0.009) from 10 mM of APAP with HMGB1 supernatant concentration at 56.6 ± 1.74 ng/ml).

### HMGB1 amplifies by itself APAP-induced hepatocyte death

**Figure 3:**
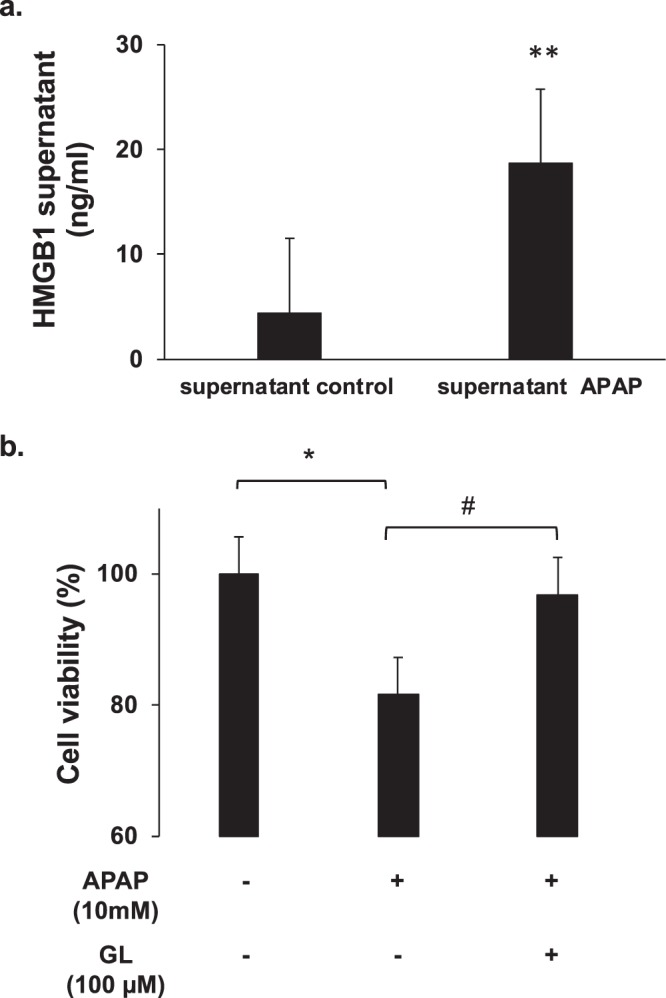
Supernatant containing HMGB1 induces hepatotoxicity. (**a**) HMGB1 levels were measured into the supernatants of HepaRG cells previously stressed by APAP (10 mM). ** p < 0.01 vs supernatant control. (**b**) Naïve HepaRG cells were exposed to supernatant of HepaRG cells previously stressed by APAP (10 mM) in presence or absence of glycyrrhizin (100 µM) for 12 hours. Cell viability was assessed by MTT method. Results are expressed as mean ± SEM. * p < 0.05 vs control. # p < 0.05 vs APAP. Experiments were reproduced three times.

**Figure 4:**
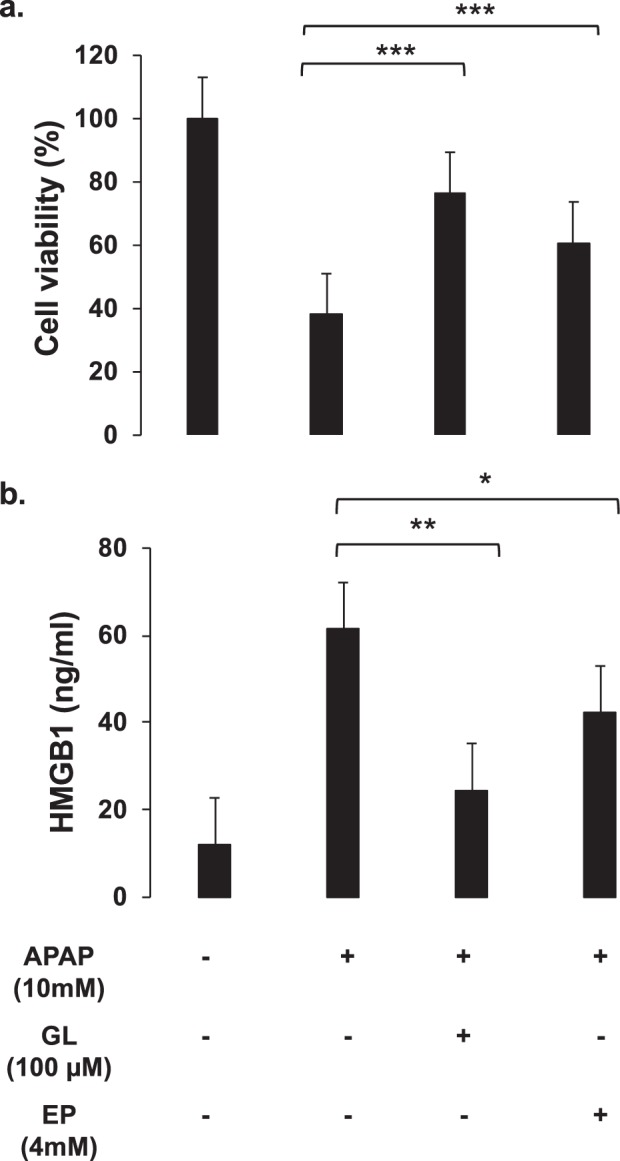
Cellular viability in presence of APAP, HMGB1 and HMGB1 inhibitors. (**a**) Cellular viability of APAP (10 mM)-exposed HepaRG cells at 24 hours. Glycyrrhizin (GL, 100 µM), ethyl pyruvate (EP, 4 mM) was added to the culture medium at the same time as APAP. Results are expressed as mean ± SEM. *vs. APAP-exposed cells, p < 0.05; ** vs. APAP-exposed cells, p < 0.01; *** vs. APAP-exposed cells (0), p < 0.001. Experiments were reproduced three times. (**b**) HMGB1 levels were measured into the supernatants of HepaRG cells exposed to APAP (10 mM), GL (100 µM) and EP (4 mM) at 24 hours. Results are expressed as mean ± SEM. *vs. APAP-exposed cells, p < 0.05; ** vs. APAP-exposed cells, p < 0.01; *** vs. APAP-exposed cells (0), p < 0.001. Experiments were reproduced three times.

Supernatant transfer experiments from APAP-exposed HepaRG cells to naïve HepaRG cells (cells non previously exposed to APAP) were performed (Suppl Fig. 1). HepaRG cells were exposed to APAP (10 mM) for 6 hours, then washed to remove cell debris and new culture medium was added for an additional 6 hours. The latter supernatant, containing cell components and HMGB1 (Fig. 3a**)**, was then added to naïve HepaRG cells for 12 hours. As shown in Fig. 4b, this supernatant induced a 18.4% mortality rate in naïve HepaRG cells (p = 0.02). However, this toxic effect is preventable by the addition of glycyrrhizin in the new culture medium (100 µM) who reduced the HepaRG cells mortality to 3.1% (p = 0.021) (Fig. 3b).

The addition of drugs known to act on HMGB1 protein, as glycyrrhizin (GL; 100μM) or ethyl pyruvate (EP; 4 mM), significantly improved HepaRG cells survival (Fig. 4a). Indeed, compared to APAP alone, addition of GL or EP increased cell viability to around 75% (p < 0.001) and 60% (p < 0.001), respectively, at 24 hours. In parallel, decreased HMGB1 concentration in the cell supernatant was observed (Fig. 4b). HMGB1 concentration dropped from 61.36 ±1.73 to 24.52 ±0.94 ng/ml (p = 0.001) after GL treatment and 42.26 ±1.26 ng/ml (p = 0.014) after EP treatment. Due to the design of the experiments, the concentration of HMGB1 in the supernatant were significantly lower than in experiments with APAP treated HepaRG (Fig. 2b). This can explain the relatively small mortality rate (18%) induced by the supernatant on naïve HepaRG cells.

**Figure 5:**
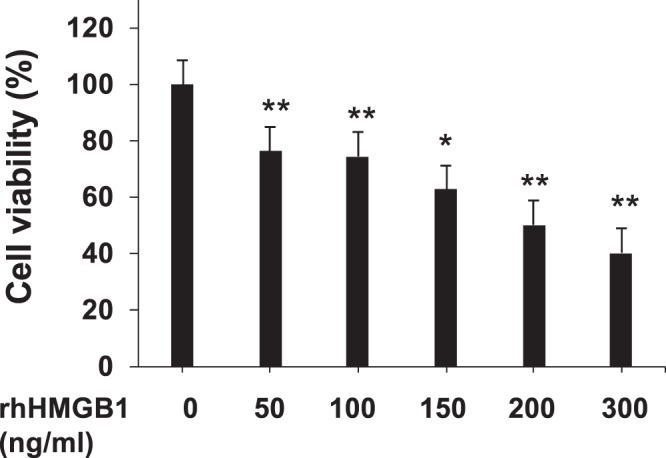
HMGB1 amplifies by itself hepatotoxicity on HepaRG cells. (**a**) Addition of recombinant human HMGB1 (rhHMGB1) to HepaRG cells exposed 24 hours to APAP (10 mM). Cell viability was evaluated after 24 hours by MTT method. Results are expressed as mean ± SEM. * vs. 0, p < 0.05. Experiments were reproduced three times.

To test a direct effect of HMGB1, HepaRG cells were exposed to increasing concentration of recombinant human HMGB1 (rhHMGB1). rhHMGB1 mediated HepaRG cell death in a dose-dependent manner as shown in Fig. 5. A significant decrease of HepaRG cells viability was observed with 50 ng/mL (p = 0.001) of rhHMGB1 (in the same range of concentrations of native HMGB1 measured in the supernatant of APAP-treated HepaRG cells in Fig. 2b).

**Figure 6:**
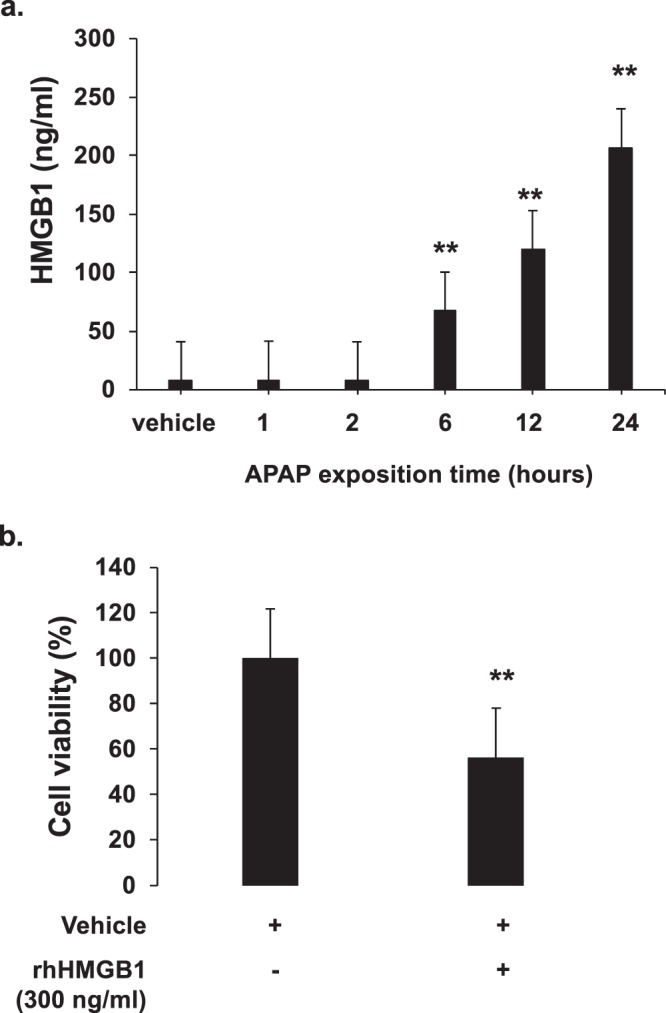
HMGB1 amplifies by itself hepatotoxicity on primary human hepatocytes. (**a**) HMGB1 levels were measured into the supernatants of HepaRG cells plated in 24 wells and treated with APAP (10 mM) at increasing time points (1, 2, 6, 12 and 24 hours). ** p < 0.01 vs vehicle. (**b**) Addition of rhHMGB1 at 300 ng/ml to primary human hepatocytes plated in 24 wells. Cell viability was evaluated after 24 hours by MTT method. Results are expressed as mean ± SEM. * vs. 0, p < 0.05. Experiments were reproduced three times.

This experiment was reproduced on primary human hepatocytes. We have previously analyzed the release of HMGB1 by HepaRG cells as a function of APAP exposure time (Fig. 6a). Thereafter, rhHMGB1 at 300 ng/ml was added during 24 hours and cell viability was assessed. As shown in Fig. 6b, rhHMGB1 also induced significant cell death in primary human hepatocytes with a drop of cell viability to 56.41 ± 3.16% (p = 0.001).

### HMGB1 and APAP induce hepatocytes necroptosis by RIPK3-dependent pathway

**Figure 7:**
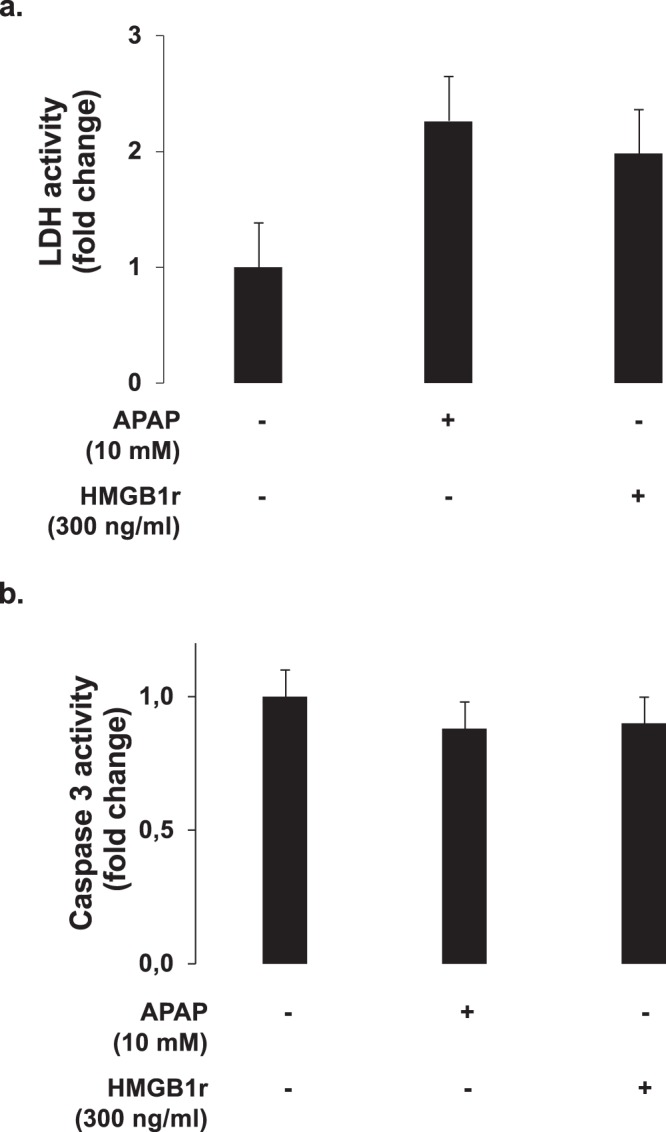
HMGB1 and APAP induce hepatocytes necrosis but not apoptosis. (**a**) LDH activity was measured after APAP (10 mM) or rhHMGB1 (300 ng/ml) treatment of HepaRG cells for 24 hours. (**b**) Caspase-3 activity after APAP (10 mM) or rhHMGB1 (300 ng/ml) treatment of HepaRG cells for 24 hours. Results are expressed as mean ± SEM. * vs. unexposed cells (0), p < 0.05; ** vs. unexposed cells (0), p < 0.01; *** vs. unexposed cells (0), p < 0.001. Experiments were reproduced three times.

**Figure 8:**
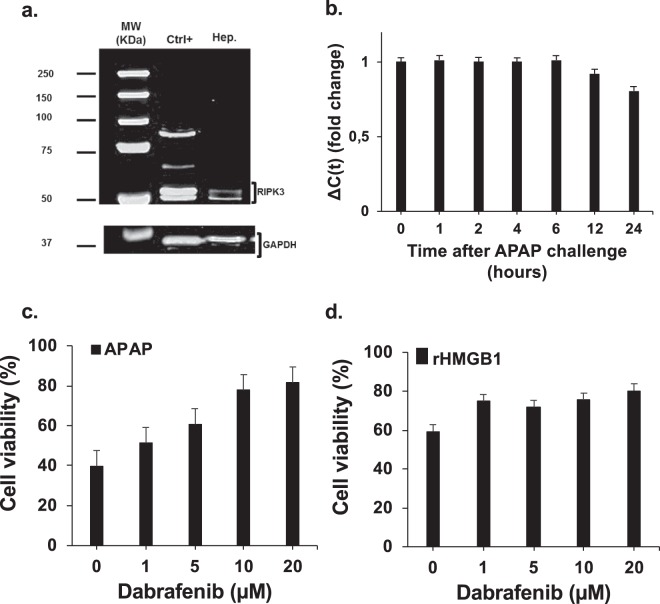
HMGB1 and APAP induce hepatocytes necrosis by RIPK3-dependent pathway. (**a**) Western Blot was performed to confirm the expression of RIPK3 by HepaRG cells. Protein extracts were prepared by lysis of HepaRG cells in Laemmli Buffer (mouse cerebellum protein lysate was used as positive control). The same protein extract was used for all the experiments. GAPDH was used as a loading control. Ctrl + , mouse Cerebellum; Hep., HepaRG cells. (**b**) Using qPCR, we evaluated the profile of RIPK3 mRNA expression in HepaRG cells exposed to APAP at different time points. β-actin gene was used as housekeeping gene. (**c**) and (**d**) Dabrafenib, RIPK3 inhibitor, at the indicated concentrations (0–20 μM) was incubated with HepaRG cells one hour prior exposition to APAP (10 mM) or rhHMGB1 (300 ng/ml). Cellular viability was measured by MTT method after 24 hours.

**Figure 9:**
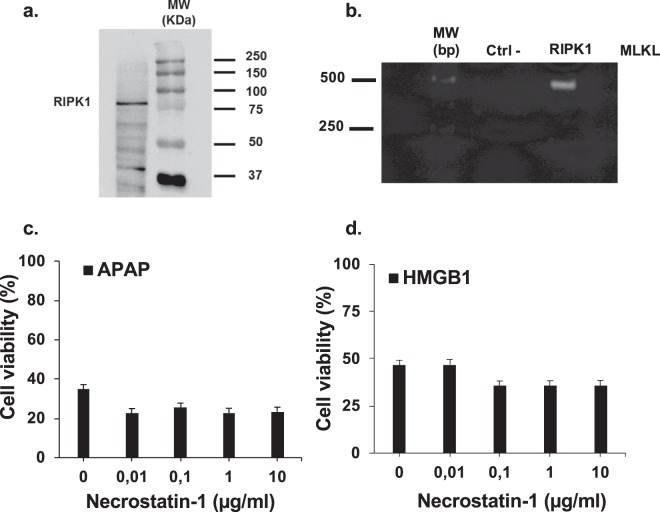
HMGB1 and APAP induce hepatocytes necrosis by RIPK1-independent pathway. (**a**) Western Blot was performed to confirm the expression RIPK1 by HepaRG cells. The same protein extract was used for all the experiments. GAPDH was used as a loading control. Ctrl + , mouse Cerebellum; Hep., HepaRG cells. (**b**) The expression of RIPK1 was assessed by classical PCR. β-actin was used as positive control. (**c**) and (**d**) Necrostatin-1, RIPK1 inhibitor, at the indicated concentrations (0–10 μg/ml) was incubated with HepaRG cells 1 hour prior to exposure to APAP (10 mM) or rhHMGB1 (300 ng/ml). Cellular viability was measured by MTT method after 24 hours. Results are expressed as mean ± SEM. * vs. 0, p < 0.05.

To understand how HMGB1 acts on HepaRG cells, we explored the potential pathway involved in APAP and rhHMGB1-induced cell death. Significant lactate dehydrogenase (LDH) release was observed after addition of rhHMGB1 (p < 0.001), as well as APAP (p < 0.001), in absence of significant caspase-3 activation as shown in Figs. 7a and b, respectively. These results suggest that necrosis represent the main death process in our model. However, different forms of regulated caspase-independent cell death have been described and necroptosis, which requires receptor interacting protein kinases (RIPK), is one of these regulated mechanisms. As demonstrated in the literature, RIPK3 and RIPK1 are implicated in the necroptosis pathway and the expression of these two proteins by HeparRG cells was confirmed at both protein (Figs. 8a and 9a**)** and mRNA levels (Figs. 8b and 9b).

In order to investigate if necroptosis is implicated in our model, inhibitors of RIPK3 and RIPK1 were used. HepaRG cells were pretreated 1 hour with inhibitors before addition of APAP or rhHMGB1 and cell viability was assessed at 24 hours. The addition of dabrafenib, a potent inhibitor of RIPK3 kinase activity, rescued significantly both APAP- and rhHMGB1-mediated HepaRG cell death in a dose-dependent manner (Fig. 8c,d). On the contrary, the addition of necrostatin-1, a selective inhibitor of RIPK1 kinase activity, to HepaRG cells had no significant effect on APAP- and rhHMGB1-mediated HepaRG cell death (Fig. 9c,d).

**Figure 10:**
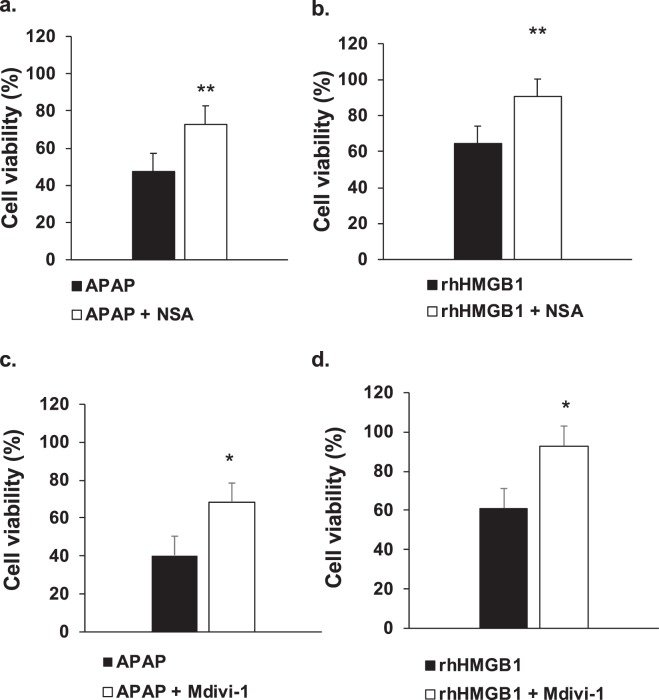
HMGB1 and APAP induce hepatocytes necrosis by RIPK3-dependent pathway. (**a**) and (**b**) Necrosulfonamide (NSA; 2.5 μM), a MLKL inhibitor, was incubated with HepaRG cells one hour prior to exposure to APAP (10 mM) or rhHMGB1 (300 ng/ml) and cellular viability was measured after 24 hours by MTT method. (**c**) and (**d**) Mdivi-1 (50 μM), a Drp-1 inhibitor, was incubated with HepaRG cells one hour prior to exposure to APAP (10 mM) or rhHMGB1 (300 ng/ml) and cellular viability was measured after 24 hours by MTT method. Results are expressed as mean ± SEM. * vs. APAP p < 0.05; ** vs. APAP, p < 0.01.

Necroptosis requires RIPK3-mediated activation of mixed lineage kinase domain-like protein (MLKL) which induces necrotic membrane rupture^21,22^. Alternatively, RIPK3 can induce mitochondrial dynamin-related protein 1 (Drp1) translocation and fission^23^. Inhibitors were added 1 hour prior APAP or rhHMGB1 and cell viability was assessed at 24 hours. Significant reduction of APAP-mediated cell death was observed in Fig. 10a after addition of NSA, an MLKL inhibitor, at 2.5 μM (p = 0.025). Similarly, rhHMGB1-mediated cell death was also reduced as observed in Fig. 10b (p = 0.045). If an inhibitor of Drp1, Mdivi-1, was added at 50 μM to HepaRG cells, significant reduction of both APAP- and rhHMGB1-mediated HepaRG cell death was observed as shown in Fig. 10c (p = 0.004) and Fig. 10d (p = 0.045), respectively.

### HMGB1 induces hepatocyte necroptosis through TLR4 adaptor TRIF pathway

After the demonstration of RIPK3 implication in our model, we explored which signaling pathway is involved upstream.

**Figure 11:**
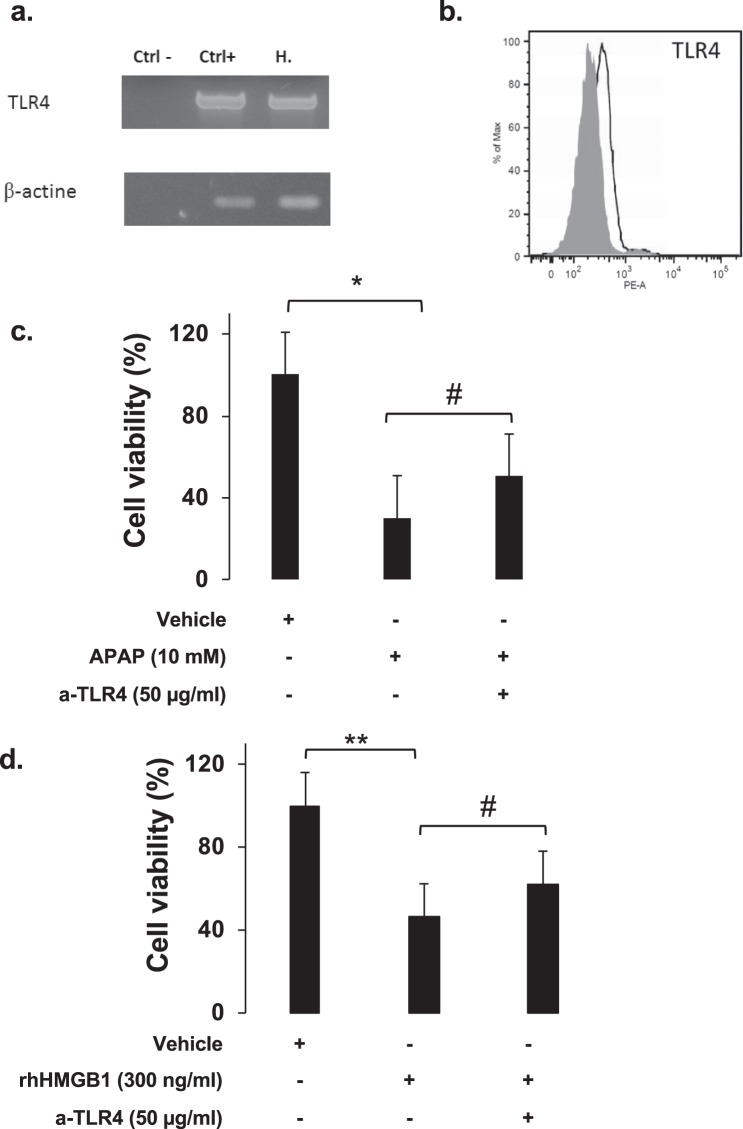
HMGB1 induces hepatocyte necroptosis through TLR4 receptor. (**a**) mRNA expression of TLR4 was confirmed by RT-PCR in HepaRG cells. β-actin was used as housekeeping gene. (**b**) HepaRG protein expression was evaluated by flow cytometry analysis and the results showed that HepaRG cell express TLR4. (**c**) Neutralizing monoclonal antibody to TLR4 at 50 μg/ml was added to HepaRG cells 1 hour prior APAP. Cellular viability was assessed by MTT method 24 hours after APAP (10 mM). Results are expressed as mean ± SEM. * vs. ctrl p < 0.05; # vs. APAP, p < 0.05. Experiments were reproduced three times. (**d**) Neutralizing monoclonal antibody to TLR4 at 50 μg/ml was added to HepaRG cells 1 hour prior rhHMGB1 (300 ng/ml). Cellular viability was assessed by MTT method 24 hours after rhHMGB1 addition. Results are expressed as mean ± SEM. ** vs. ctrl p < 0.01; # vs. HMGB1, p < 0.05. Experiments were reproduced three times.

It is well known that extracellular HMGB1 acts through multiple receptors, including Toll-like receptors (TLR4). HepaRG cells expressed TLR4 at mRNA (Fig. 11a) and protein (Fig. 11b) levels. To investigate the role of this receptor, we used neutralizing monoclonal antibody against TLR4 (a-TLR4). HepaRG cells were pretreated 1 hour with a-TLR4 at 10 μg/mL and cell viability was assessed 24 hours after APAP or rhHMGB1 addition. A significant decrease of HepaRG cells death after APAP (p = 0.020) or rhHMGB1 (p = 0.046) exposition was observed as shown in Fig. 11c and Fig. 11d, respectively.

**Figure 12:**
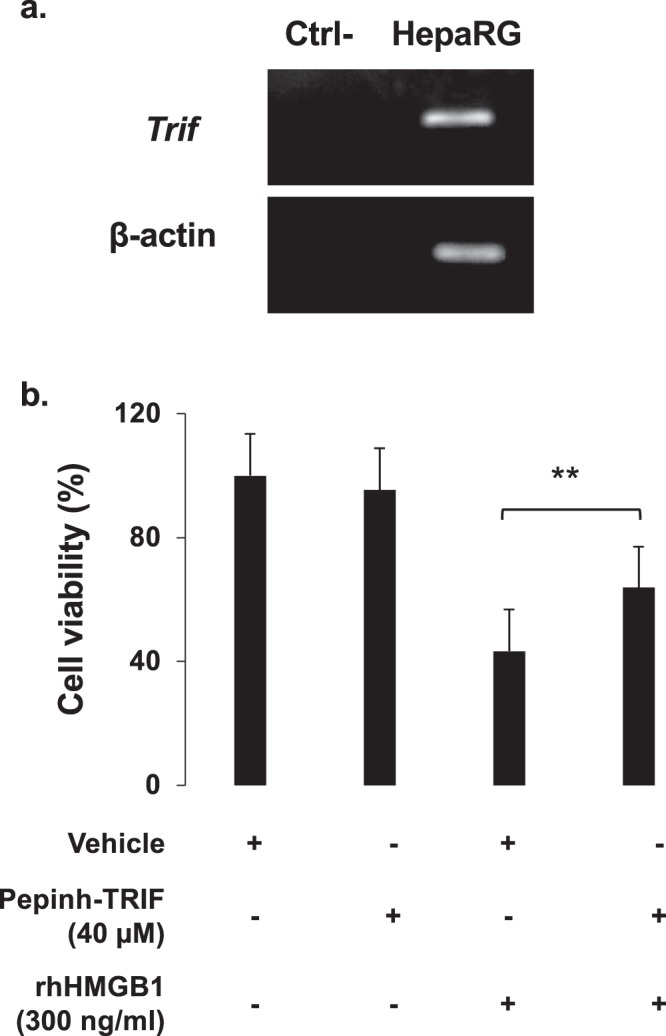
HMGB1 induces hepatocyte necroptosis through TLR4 adaptor TRIF pathway. (**a**) mRNA expression of TRIF was confirmed by RT-PCR in HepaRG cells. β-actin was used as housekeeping gene. (**b**) *In vitro*, HepaRG cells were exposed to a TRIF inhibitor peptide (Pepinh-TRIF, 40 µM) or to a control peptide (Pepinh-Control, 40 µM), 6 h prior rhHMGB1 (300 ng/ml) and cell viability was evaluated by MTT after 24 h. Results are expressed as mean ± SEM. ** vs. HMGB1, p < 0.01.

Signaling by TLRs involves cytosolic Toll/Interleukin-1 receptor (TIR) domain-containing adaptor, such as TRIF. A noncanonical necroptosis has been described in which TRIF activates RIPK3 without the requirement of RIPK1^24^. Expression of TRIF at mRNA level by HepaRG cells was confirmed in Fig. 12a. Using a peptide inhibitor (PepInh), the potential implication of TRIF has been investigated in our model. HepaRG cells were pretreated PepInh-TRIF at 40 µM 6 hours prior rhHMGB1 (300 ng/ml). Cell viability was assessed 24 hours after rhHMGB1 addition and improved HepaRG cell viability by 20.27% (p = 0.009) was observed as shown in Fig. 12b.

## Discussion

Our study suggests a new explicative mechanism for the propagation of hepatocyte death during APAP-induced liver injury. First, our experiments reveal that released HMGB1 from dying hepatocytes contributes by a feedforward circuit to the amplification of hepatocyte necrosis. Actually, many DAMPS are probably released in the supernatant of APAP-treated-cells and not only HMGB1. HMGB1 protein is known to bind to other DAMPS to promote various signaling pathways^25^. We have not explored this part. However, we have been able to prove the existence of HMGB1 in the supernatant exposed to naïve cells (Fig. 4a). Second, we observed that HMGB1 induces hepatocyte necrosis by RIPK3-dependent pathway suggesting necroptosis implication. Third, we observed that released HMGB1 directly acts on neighboring hepatocytes through TLR4-TRIF pathway.

In accordance with others, we have confirmed that the administration of GL reduces the severity of APAP-induced liver injury in murine model^13,26,27^. In parallel, in mice treated with GL, we observed a decrease in the release of HMGB1 from hepatocytes: immunostaining quantification and serum HMGB1 levels were measured. GL is known to have many effects including inhibition of HMGB1. Based on these results, the present study was initiated to assess whether HMGB1 released from dying hepatocytes could act on neighboring hepatocytes independently of inflammatory cells. We confirmed this hypothesis *in vitro* using cultured HepaRG cells. As expected, and without interference with APAP bioactivation (Suppl Fig. 4**)**, we observed that the addition of GL or EP reduced APAP-induced HepaRG cell death, suggesting that HMGB1 contributed to the APAP-induced death process.

We demonstrated^28^ that hepatocytes expressed the cognate HMGB1 receptors TLR2, TLR3, TLR4, and TLR9 allowing for a direct response to released HMGB1 and that HMGB1 by itself can induce cell death. Several previous experiments using knock-out mice have suggested that TLR2, TLR3, TLR4, TLR9, and RAGE could contribute to APAP-induced liver injury although some controversy remains pertaining to possible dual or opposite effects according to which cell types are studied^29–31^. We focused on TLR4 receptor in our experiments. Inhibition of TLR4 receptor using blocking antibody, prevents APAP induced HepaRG cells death. These results suggested the implication of TLR4 receptor in the hepatotoxicity process induced by APAP.

We observed that both APAP and HMGB1-induced HepaRG death was characterized by LDH release without implication of caspase-3. Different forms of regulated necrosis have been described and to distinguish RIPK-dependent necrosis from other forms, the term necroptosis is used. We know that the activation of RIPK1 and RIPK3 is required for the activation of the necrosome. In this study, we investigated the inhibition of each one. We showed that RIPK3 inhibition by dabrafenib significantly attenuated APAP and rhHMGB1-induced HepaRG cell death without interference with APAP bioactivation (Suppl Fig. 4**)**. Previous studies have shown that RIPK3 genetic ablation or inhibition by dabrafenib partially protected mice from APAP-induced liver injury although this observation was not reproduced in RIPK3−/− mice^32–34^. The reasons of these discrepancies are uncertain. In the other hand, the inhibition of RIPK1 by necrostatin-1 is still controversial: protective^35^ and deleterious^36,37^ roles have been reported. In our *in vitro* experiments, inhibition of RIPK1, had no impact on rhHMGB1- and APAP-induced cell death in our experiments. Taken together, our *in vitro* data support a model in which HMGB1 causes hepatocyte cell death via RIPK3-dependent, RIPK1-independent, noncanonical activation of necroptosis.

As previously mentioned, hepatocytes express a part of TLR repertoire which is able to bind HMGB1. An intracellular adaptor implicated in TLR signaling, called TRIF, have a Receptor interaction protein kinase (RIPK) homology interaction motif (RHIM) which activates in some instances, in cooperation with RIPK3, a noncanonical programmed cell necrosis^24^. Interestingly, we showed that inhibition of TRIF by Pepinh-TRIF significantly attenuated rhHMGB1-induced HepaRG cell death. In addition, we previously observed that *Trif* mutant mice are partially protected against APAP-induced liver damage^28^.

In conclusion, we observed *in vitro* that, in APAP-induced hepatotoxicity, released HMGB1 from damaged hepatocytes contributed to the propagation of necrosis by a direct effect on neighboring hepatocytes in a feed-forward circuit. Our results give new insights in the acetaminophen toxicity by suggesting that HMGB1 amplifies by itself the hepatocyte necrosis trough TLR4 -TRIF- RIPK3-dependent pathway.

## Materials and Methods

### Antibodies, cells, and other reagents

Human antibodies were obtained for TLR4 from BD Pharmingen (San Diego, USA), RIPK1 and MLKL from Proteintech (Rotorua, New Zealand), RIPK3 from Thermo Fisher Scientific (Waltham, USA) and HMGB1 from Abcam (Cambridge, UK). HepaRG cells were purchased from Invitrogen and primary human hepatocytes from ThermoFisher Scientific (Waltham, USA). Acetaminophen (APAP), glycyrrhizin (GL), ethyl pyruvate (EP), necrostatin-1 (Nec-1), tranylcypromine and ketoconazole were purchased from Sigma-Aldrich (Darmstadt, Germany). Dabrafenib was obtained from Selleck. Chemicals (Munich, Germany). Neutralizing IgG monoclonal antibody to human TLR4/MD2, TRIF inhibitory peptide (Pepinh-TRIF) and control peptide (Pepinh-Control) were purchased from InvivoGen (Toulouse, France). To ascertain that used drugs do not interfere with APAP bioactivation, CYP2E1 and CYP3A4 activity were evaluated using Vivid® CYP2E1 Blue Screening Kit and Vivid® CYP3A4 Green Screening Kit (ThermoFisher Scientific, Waltham, MA, USA) **(**Suppl Figs. 2, 3**)**.

### Assessment of glutathione

Assessment of glutathione (GSH) levels were performed using BIOXYTECH GSH-400 colorimetric assay kit and following the manufacturer’s protocol (OxisResearchTM, USA). The enzyme concentration was expressed as nmol of enzyme per milligram of protein. The protein concentration was evaluated using Quick Start Bradford Protein Assay (Bio-Rad, USA). Bovine serum was used as a standard.

### Cell culture

HepaRG cells (Invitrogen, California, US) were cultured in growth medium (William’s E medium with Glutamax I, supplemented with 100 U/ml penicillin-streptomycin, 5 µg/ml human recombinant insulin and 0.5 µM hydrocortisone hemisuccinate) at 37 °C with 5% CO2. HepaRG cells were cultured in 24-well plates or in 96-well plates depending on the protocol used. In Figs. 4 and 6, the HepaRG cells were seeded on 24-well plate and exposed to APAP for only 6 hours; the time required for HepaRG cells to become stressed (minor inducer of death). Cells were then washed to remove cell debris and new culture medium was added for an additional 6 hours without APAP. The latter supernatant was added to the naïve cells for 12 hours. In this experiment, the aim was to investigate whether compounds (DAMPs) released by stressed cells was capable of stress/killing naïve cells. In all other experiments performed, HepaRG cells were seeded on 96-well plate and exposed to APAP for 24 hours (major inducer of death).

For HepaRG cells differentiation, cells were cultured in the medium for 2 weeks and then in presence of 2% dimethyl sulfoxide (DMSO) for 2 more weeks. Thawing, seeding, culture, differentiation and maintenance of HepaRG cells for toxicity study were performed as described in the HepaRG^TM^ Cell User Guide provided by the firm and as previously described in the literature^18^.

Primary human hepatocytes (HU1539, Life Technologies, Waltham, MA, USA) were thawed and plated in collagen I-coated 24-wells plates following the manufacturer’s protocol. Cells were cultured 24 hours in incubation medium (Williams’ E medium (1×, no phenol red) and Hepatocyte Maintenance Supplement Pack (serum free)) at 37 °C with 5% CO2. After that, primary human hepatocytes were exposed to rhHMGB1 for 24 hours at 300 ng/ml (concentration measured in the supernatant of HepaRG cells APAP-treated as shown in Fig. 6a).

### Cell viability assay

Cell viability was evaluated using 3-(4,5-dimethylthiazol-2-yl)-2,5-diphenyltetrazolium bromide (MTT). Briefly, 10 µL of MTT diluted in 90 µL of culture medium were added to each well and incubated for 3 hours at 37 °C. After removing MTT solution, 100 µL of hydrochloric acid/isopropanol was added to each well for 10 min. The absorbance was read at 540 nm. The relative cell viability (%) related to control wells containing cells and culture medium without treatment was calculated by ((A) test/(A) control) × 100.

### Cell death process

Lactate dehydrogenase (LDH) activity was determined using the Pierce LDH Cytotoxicity Assay Kit (ThermoFisher Scientific, Belgium) per the manufacturer’s instructions. Caspase-3 activity was quantified using the Caspase-3 colorimetric assay (R&D Systems, Minnesota, USA) per the manufacturer’s instructions.

### Enzyme-linked immunosorbent assay (ELISA)

HMGB1 concentrations in the supernatant of cultured HepaRG cells was measured by a sandwich-enzyme immunoassay (IBL International GmbH, Hamburg, Germany) following the manufacturer’s protocol.

Trace quantities of acetaminophen in the supernatant of cultured HepaRG cells was measured using Neogen Corporation’s test kit (Lexington, KY, USA). It operates based on competition between the drug in the sample and the drug-enzyme conjugate for a limited number of antibody binding sites. The presence of bound drug-enzyme conjugate is recognized by the addition of K-Blue® Substrate. The extent of color development at 450 nm is inversely proportional to the amount of drug in the sample.

### Flow cytometry analysis

After washing and counting, HepaRG cells (5×10^5^ cells/50 µl) were incubated for 20 min at 4 °C with 10 µl (dilution 1:5) of FC Blocking Reagent (MACS®). After washing, cells were incubated 30 min at 4 °C with indicated antibody or the corresponding isotype controls in PBS/serum/0.01% azide to reduce the nonspecific uptake of mAbs.

The following antibody was used: anti-TLR4-PE (TF90). Samples were acquired using a BD FACSCanto II (BD Bioscience, San Diego, CA, USA) flow cytometer and data were analyzed using FlowJo software (V10.0.7).

### Immunofluorescence

Immunofluorescence of HMGB1 was performed on HepaRG cells untreated. Briefly, cells were fixed with paraformaldehyde 1%, permeabilized with triton 0,4%, stained for HMGB1 (green) and DAPI (blue) and imaged by confocal microscopy (magnification x600).

### Protein extraction and western blot

Protein extracts were prepared by lysis of HepaRG cells in Laemmli Buffer (mouse cerebellum protein lysate was used as positive control). As previously described, protein concentrations were measured by chromatography using bovine serum albumin as a standard^38^. Then, proteins were separated by 10% SDS-PAGE followed by transfer to a nitrocellulose membrane. After blocking for 1 h at room temperature with blocking buffer (Li-Cor Biosciences, Lincoln, NE, USA), blot was incubated with antibody against RIPK3 (1:1000), RIPK1 (1:500), or MLKL (1:1000). The membrane was washed three times with PBS/Tween 0.1% and incubated with a DyLight800-conjugated goat anti-rabbit antibody at a 1:15000 dilution (ThermoFisher Scientific, Belgium) or with ECL anti-rabbit IgG Horseradish Peroxidase conjugated antibody at a 1:2000 dilution (GE Healthcare, UK). Finally, proteins were visualized with a fluorescent Western scanning, Odyssey Infrared Imaging System (Li-Cor Biotechnology, USA) or with a charge-coupled device (CCD) camera (Fusion FX5, Vilber Lourmat, France) per the manufacturer’s instructions.

### RNA extraction and RT-PCR

RNA was extracted using a High Pure RNA Tissue kit (Roche Diagnostics, Brussels, Belgium). Quantification of mRNA was performed using a 2-step real-time reverse-transcriptase polymerase chain reaction (LightCycler, Roche Diagnostics). The relative expression of the gene of interest was calculated against the β-actin gene. For classical RT-PCR, preparation of cDNA and PCR were performed using standard procedures. The sequences of primers are detailed in the Table 1.

**Table 1:** The list and sequences of primers used for PCR analysis.

	Primer	Species	Direction	Sequences
Classic PCR	β-actin	Human	Forward	5′-GGATGCAGAAGGAGATCACTG-3′
			Reverse	5′-CGATCCACACGGAGTACTTG-3′
	TLR4	Human	Forward	5′- TCCCTCCAGGTTCTTGATT-3′
			Reverse	5′ - GTCTCTGTAGTGAAGGCA-3′
	TRIF (TICAM-1)	Human	Forward	5′-GGATCCCTGATCTGCTTGGG-3′
			Reverse	5′-CCTCATCCTGAAGTTCCCCC-3′
	RIPK1	Human	Forward	5′-ATTCATGACGGCGCTCAGATTT-3′
			Reverse	5′-GTTCCAAAGCCATGTGAGCTATA-3′
Real Time PCR	β-actin	Human	Forward	5′-TGAAGTACCCCATCGAGCAC-3′
			Reverse	5′-GGTAGTCAGTCAGGTCCCGG-3′
			Probe	5′-CCAGATTTTCTCCATGTCGTCCCA-3′
	RIPK3	Human	Forward	Confidential Roche Diagnostics
			Reverse	
			Probe	

### Statistical analysis

Statistical analyses were performed using SPSS Statistics 18.0 (Chicago, IL, USA). Differences between groups were assessed using the Kruskal-Wallis, the Mann-Whitney *U* or Student’s *t* test, as appropriated. Rates of mouse survival were assessed using the Kaplan-Meier method and compared using the log-rank test. Results are expressed as mean ± SEM. A p-value of <0.05 was considered as statistically significant.

## Supplementary information

Supplementary Information.
