# Supplementary material for: New insights in acetaminophen toxicity: HMGB1 contributes by itself to amplify hepatocyte necrosis in vitro through the TLR4-TRIF-RIPK3 axis
Source: Sci Rep. 2020 Mar 27;10:5557. doi: 10.1038/s41598-020-61270-1 (PMC7101425; doi:10.1038/s41598-020-61270-1)
Supplement: Supplementary file 1 — Supplementary Information. [file 41598_2020_61270_MOESM1_ESM.pdf]

New insights in acetaminophen toxicity: HMGB1 contributes by itself to amplify hepatocyte necrosis in vitro through the TLR4-TRIF-RIPK3 axis.

***Charlotte Minsart[1]**, Claire Liefferinckx[1], Arnaud Lemmers[1,2], Cindy Dressen[3], Eric Quertinmont[1], Isabelle Leclercq[4], Jacques Devière[1,2], Richard Moreau[5,6,7], Thierry Gustot[1,2,6,7].

Suppl Fig 1

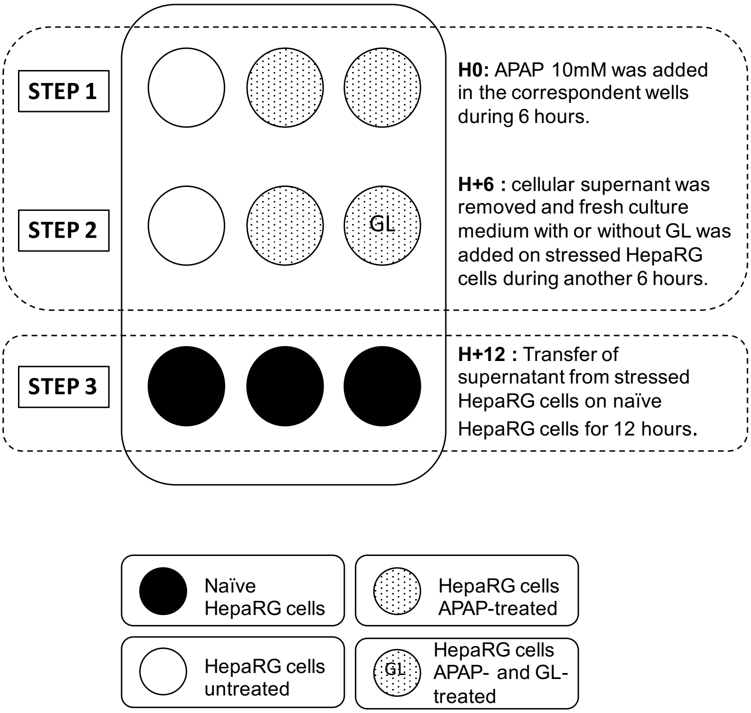

Suppl Fig 1. Schematic explanation of the method used for the "supernanant transfer experiment".

HepaRG cells were cultured in 24-well plate and seeded at a density of $0{,}60 \times 10^6$ per well following manufacturer's instructions (Invitrogen, California, US).

Step one: HepaRG cells are stressed with APAP (10 mM) for 6 hours . Step two: HepaRG cells are gently washed to remove cell debris and new culture medium was added for further 6 hours with and without glycyrrhizin. Step three: This last medium was then transferred on naïve HepaRG cells for 12 hours.

New insights in acetaminophen toxicity: HMGB1 contributes by itself to amplify hepatocyte necrosis in vitro through the TLR4-TRIF-RIPK3 axis.

***Charlotte Minsart[1]**, Claire Liefferinckx[1], Arnaud Lemmers[1,2], Cindy Dressen[3], Eric Quertinmont[1], Isabelle Leclercq[4], Jacques Devière[1,2], Richard Moreau[5,6,7], Thierry Gustot[1,2,6,7].

Suppl Fig 2

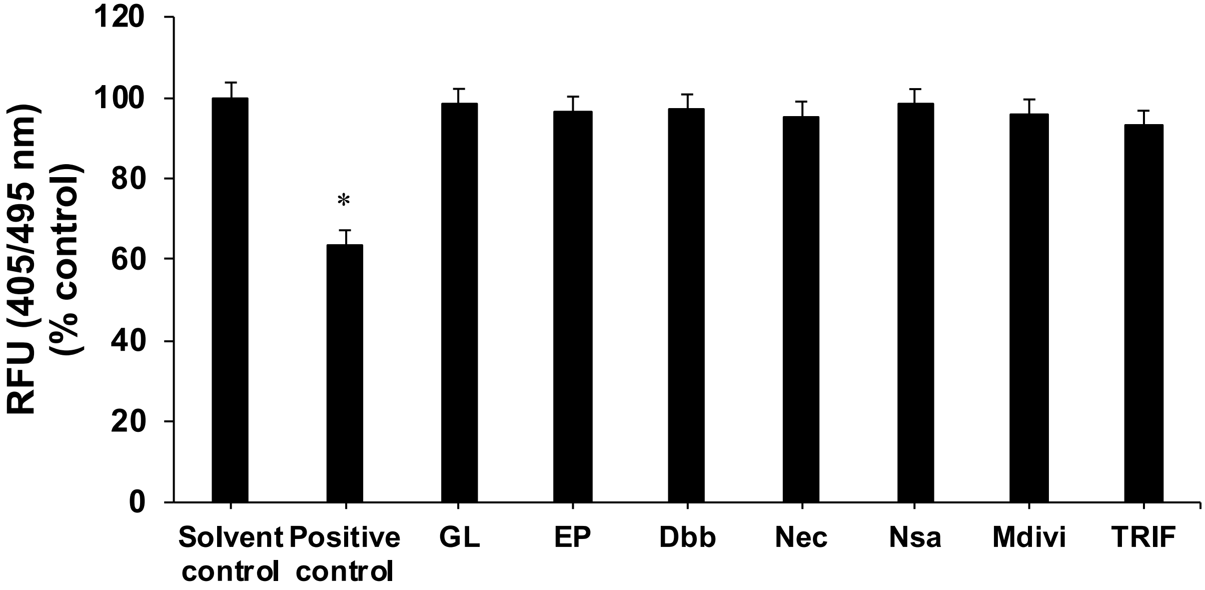

Suppl Fig 2. CYP2E1 activity is not influenced by drugs used in our experiments.
Because pharmaceuticals drugs were used in this study, it was necessary to exclude interference with APAP metabolism. For this reason, the cytochrome P450 (CYP)2E1 activity was assessed using Vivid® CYP450 Screening Kits and following the manufacturer's protocol (ThermoFisher Scientific, Waltham, MA, USA). Briefly, using 7-ethoxy-methyloxy-3-cyanocoumarin (EOMCC) as a fluorogenic substrate and CYP450 BACULOSOMES® (microsomes prepared from insect cells expressing a human CYP450 isozyme), we evaluated the effects of different drugs on cytochrome P450 (CYP)2E1 activity : glycyrrhizin (GL;100µM), ethyl-pyruvate (EP; 4mM), dabrafenib (Dbb; 20µM), necrostatin-1 (Nec; 1µg/ml), necrosulfonamide (2,5 µM), Mdivi (50 µM) and pepinh-TRIF (40µM).
The results demonstrated the absence of interaction between drugs used and CYP2E1 activity. Tranylcypromine (TCP, 1mM) was used as a positive control. *P<0,05 vs solvent control. Abbreviation: RFU, Relative Fluorescence Units.

New insights in acetaminophen toxicity: HMGB1 contributes by itself to amplify hepatocyte necrosis in vitro through the TLR4-TRIF-RIPK3 axis.

***Charlotte Minsart[1]**, Claire Liefferinckx[1], Arnaud Lemmers[1,2], Cindy Dressen[3], Eric Quertinmont[1], Isabelle Leclercq[4], Jacques Devière[1,2], Richard Moreau[5,6,7], Thierry Gustot[1,2,6,7].

Suppl Fig 3

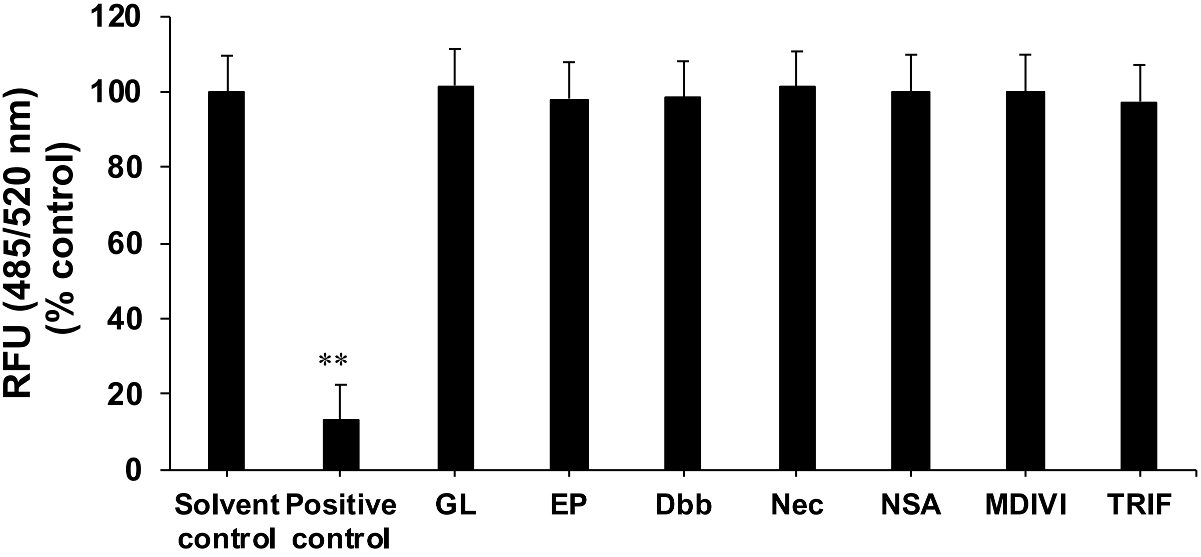

Suppl Fig 3. CYP3E4 activity is not influenced by drugs used in our experiments.
Because pharmaceuticals drugs were used in this study, it was necessary to exclude interference with APAP metabolism. For this reason, the cytochrome P450 (CYP)3A4 activity was assessed using Vivid® CYP450 Screening Kits and following the manufacturer's protocol (ThermoFisher Scientific, Waltham, MA, USA). Briefly, using 7-ethoxy-methyloxy-3-cyanocoumarin (EOMCC) as a fluorogenic substrate and CYP450 BACULOSOMES® (microsomes prepared from insect cells expressing a human CYP450 isozyme), we evaluated the effects of different drugs on cytochrome P450 (CYP)3A4 activity : glycyrrhizin (GL; 100 µM), ethyl-pyruvate (EP; 4mM), dabrafenib (Dbb; 20µM), necrostatin-1 (Nec; 1 µg/ml), necrosulfonamide (2,5 µM), Mdivi (50 µM) and pepinh-TRIF (40 µM).
The results demonstrated the absence of interaction between drugs used and CYP3A4 activity. Ketoconazole (10 mM) was used as a positive control. ** p<0,01 vs solvent control. Abbreviation: RFU, Relative Fluorescence Units.

New insights in acetaminophen toxicity: HMGB1 contributes by itself to amplify hepatocyte necrosis in vitro through the TLR4-TRIF-RIPK3 axis.

***Charlotte Minsart[1]**, Claire Liefferinckx[1], Arnaud Lemmers[1,2], Cindy Dressen[3], Eric Quertinmont[1], Isabelle Leclercq[4], Jacques Devière[1,2], Richard Moreau[5,6,7], Thierry Gustot[1,2,6,7].

Suppl Fig 4

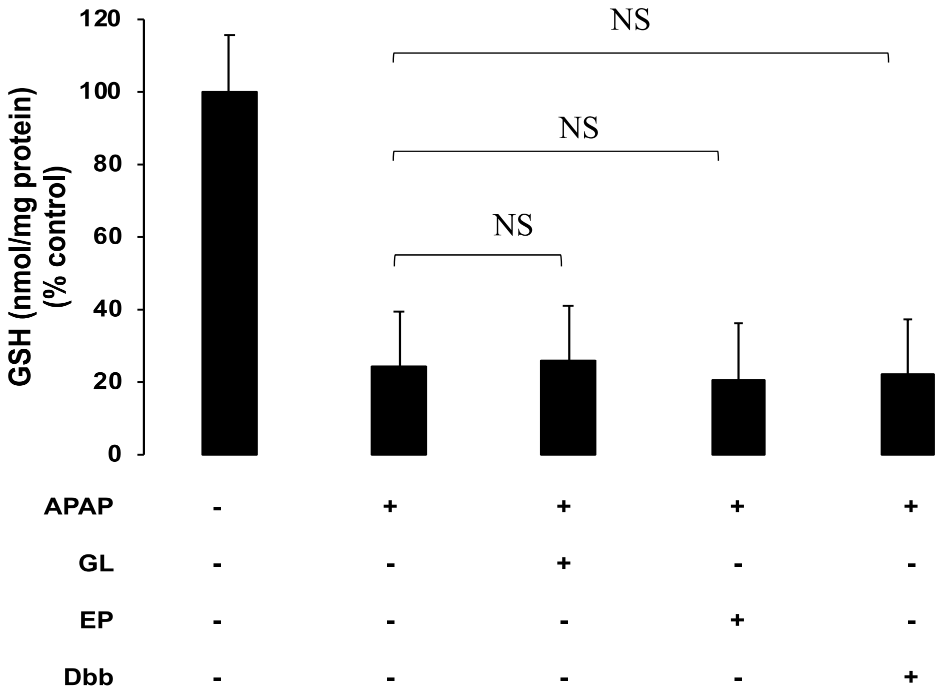

Suppl Fig 4. The APAP-induced decrease of GSH level was similar with or without the different treatments suggesting an absence of interference with APAP bioactivation.

HepaRG cells were treated with APAP (10 mM) and/or different drugs for 24 hours. . GL and EP were added at the same of APAP and Dbb 1 hour before APAP exposition. GSH levels were measured using BIOXYTECH GSH-400 colorimetric assay kit and following the manufacturer's protocol (OxisResearchTM, USA). Drugs were added on HepaRG cells at the concentration who have demonstrated beneficial effect : glycyrrhizin (GL; 100 µM), ethyl-pyruvate (EP; 4 mM) and dabrafenib (Dbb; 20 µM). The enzyme concentration obtained is expressed as nanomoles of enzyme per milligram of protein using bovine serum as a standard. ** $p<0.01$ vs vehicle.
